# Biologically weighted LASSO: enhancing functional interpretability in gene expression data analysis

**DOI:** 10.1093/bioinformatics/btae605

**Published:** 2024-10-16

**Authors:** Sofia Mongardi, Silvia Cascianelli, Marco Masseroli

**Affiliations:** Dipartimento di Elettronica, Informazione e Bioingegneria (DEIB), Politecnico di Milano, Milan 20133, Italy; Dipartimento di Elettronica, Informazione e Bioingegneria (DEIB), Politecnico di Milano, Milan 20133, Italy; Dipartimento di Elettronica, Informazione e Bioingegneria (DEIB), Politecnico di Milano, Milan 20133, Italy

## Abstract

**Motivation:**

Feature selection approaches are widely used in gene expression data analysis to identify the most relevant features and boost performance in regression and classification tasks. However, such algorithms solely consider each feature’s quantitative contribution to the task, possibly limiting the biological interpretability of the results. Feature-related prior knowledge, such as functional annotations and pathways information, can be incorporated into feature selection algorithms to potentially improve model performance and interpretability.

**Results:**

We propose an embedded integrative approach to feature selection that combines weighted LASSO feature selection and prior biological knowledge in a single step, by means of a novel score of biological relevance that summarizes information extracted from popular biological knowledge bases. Findings from the performed experiments indicate that our proposed approach is able to identify the most predictive genes while simultaneously enhancing the biological interpretability of the results compared to the standard LASSO regularized model.

**Availability and implementation:**

Code is available at https://github.com/DEIB-GECO/GIS-weigthed_LASSO.

## 1 Introduction

New sequencing technologies have led to the creation of high-dimensional datasets that are often challenging to analyze effectively. These datasets usually include thousands of features and suffer from several issues, such as the presence of noise, “curse of dimensionality,” and multicollinearity, i.e. high correlation among the dataset variables. Notably, RNA sequencing (RNA-seq) allows for the simultaneous measurement of the expression levels of thousands of genes in a biological sample, providing a clear picture of cells’ gene activity. Such information can be leveraged to reveal disease mechanisms, identify new drug targets, or explore various fundamental biological questions. However, one of the main computational challenges when working with large gene expression datasets is the “large p, small n” paradigm. Indeed, in these datasets, the number *p* of features is significantly larger than the number *n* of available samples (p≫n), which can often lead to instability and overfitting problems when fitting predictive models. Consequently, feature selection algorithms are used to enhance model performance and interpretability, by identifying a smaller subset of features with the highest predictive and discriminative power. The most widely used feature selection approaches are usually categorized as filter, wrapper, and embedded methods. The last group of algorithms directly integrates the feature selection step into the learning process by simultaneously performing parameter estimation and feature selection. The Least Absolute Shrinkage and Selection Operator (LASSO) regularization (Tibshirani 1996) is probably the most popular example of such techniques. It performs a regularization of the model parameters, i.e. the coefficients associated with each feature, by applying a $L_{1}$ penalty and shrinking their values. It has the attractive characteristic of inducing sparsity: the coefficients of features deemed irrelevant for the predictive task are set to zero. When applied to gene expression data, LASSO and traditional feature selection approaches select genes solely based on their quantitative contribution to the predictive task, i.e. they only consider the gene expression values and their statistical significance in terms of expression behavior. However, often the statistical significance and predictive power of each selected feature cannot be translated directly to its biological relevance in gene-expression data analysis (Liu *et al.* 2005). As a result, the selected feature subsets might provide limited biological interpretability and insights. Indeed, the biological knowledge associated with each gene, defined in terms of known gene properties and relationships, is key to understanding its underlying role and mechanisms in generating the observed phenotype.

Incorporating additional knowledge in the data analysis and modeling process can potentially support data-derived hypotheses by taking advantage of previously identified relationships, avoiding spurious correlations, and linking the analysis findings to the prior existing knowledge (Bellazzi and Zupan 2007, Pasquier *et al.* 2008). Feature selection algorithms that integrate prior biological knowledge can select features based not only on their statistical significance but also on their biological relevance. The final goal is to optimize the predictive task while enhancing the biological interpretability of the results (i.e. of the features identified to represent them mostly), to potentially expedite the biological discovery and downstream analysis. Existing biological knowledge can be directly extracted from publicly available knowledge bases or repositories that store curated information, such as gene functions and interactions, biological pathways, and disease phenotypes. Popular knowledge sources include the Gene Ontology (GO) (Ashburner *et al.* 2000), the Kyoto Encyclopedia of Genes and Genomes (KEGG) (Kanehisa and Goto 2000), and the Human Phenotype Ontology (HPO) (Robinson *et al.* 2008).

Several prior knowledge base approaches for the analysis of gene expression data have been developed in recent years. Most of them rely on filtering feature selection techniques to identify the most biologically relevant and predictive genes, using a predefined metric to rank genes before performing the classification or regression task. Among these, Qi and Tang (2007) proposed to use the information gain (IG) and GO annotations of each gene to iteratively rank features and to select a subset of candidate genes based on the obtained feature ranking. Fang *et al.* (2014) used the IG as an initial filter, followed by association analysis based on GO annotations and KEGG pathways to find co-occurring genes. Papachristoudis *et al.* (2010) first selected the most discriminative genes and then added genes with the highest GO-similarity score to the selected ones. Although these approaches combine the statistical significance and the biological relevance of genes, filtering might perform a stringent feature selection that selects gene candidates with limited predictive power but strong biological relevance, potentially limiting discovery, as unknown gene functions and interactions cannot be detected.

Integrated prior knowledge approaches can overcome this limitation as the predictive power and biological relevance of genes are evaluated simultaneously. The majority of integrated approaches in literature uses a weighted LASSO regularization (Zou 2006), which considers feature-specific penalties inversely proportional to the relevance of each feature. Bergersen *et al.* (2011) proposed to incorporate prior information, in the form of correlation between the genes and external biological knowledge, into a weighted LASSO problem. Zeng *et al.* (2021), instead, used a weighted $L_{1}$ regularized regression to incorporate external meta-features that provide feature-related biological information, and developed an empirical Bayes method to learn the individual penalties. Alternatively, Yang *et al.* (2024) recently proposed a two-stage prior LASSO (TSPLASSO) method that uses cancer-related genes as prior information. These prior genes are used to identify closely related candidate genes in the first step, while in the second step, both prior and candidate genes are given as input to a logistic regression model with LASSO regularization. While all these methods highlight the advantages of using an integrated approach, they all rely on manually selected and specific prior biological information or dataset-derived information.

Here, we propose a novel one-step and embedded integrative feature selection approach that combines weighted LASSO feature selection and prior biological knowledge to improve the biological interpretability of regression and classification results. Our approach is based on a new score of biological relevance that summarizes the prior existing information for each gene across one or multiple different biological knowledge bases. This score can be directly incorporated into the optimization function of a prediction algorithm with LASSO regularization to individually penalize genes based on their estimated biological relevance. By using such a score, our proposed approach provides a more flexible and general strategy for prior-knowledge-guided feature selection that does not require any domain-specific external information to be specified. The goal is to find a trade-off between the predictive power of each selected feature, with respect to the prediction task, and its relevance in terms of biological interpretability. Results from the performed experiments highlight that our proposed approach can identify the most predictive genes while simultaneously increasing the biological interpretability of the results, with respect to the standard LASSO regularized model and other popular feature selection algorithms widely used in gene expression data analysis.

## 2 Materials and methods

In the following section, we describe the methodologies used in this study. Our proposed score of biological relevance is first introduced, followed by a detailed description of the LASSO and weighted LASSO regularized models, together with the extension of the latter that incorporates our proposed score.

### 2.1 Prior knowledge score

Assume to have a dataset of *q* genes and a set of *l* knowledge bases of interest, each represented as an ontology, with a controlled vocabulary structured as a directed acyclic graph (DAG) describing the relationships among its $n_{l}$ terms. Also, assume that for each gene *g* in the dataset, we can retrieve the most specific terms the gene is associated with for each considered knowledge base. Then, we can leverage the DAG structure of each knowledge base to unfold each of such specific terms and extract its ancestors at any depth level of the DAG, considering all or only a subset of the existing relationships. Hence, for each gene in the dataset, we can obtain an unfolded list of terms that are associated with that gene, i.e. all its annotations in the considered knowledge bases.

The obtained lists of annotations can be used to build a binary annotation matrix $B\in R^{q\times \sum_{k=1}^{l}n_{k}}$ with features (genes) along the rows, annotation terms along the columns, and binary values (1/0) in each entry to indicate whether a gene is annotated with a given term or not. Starting from this annotation matrix, a weighted annotation matrix $W\in R^{q\times \sum_{k=1}^{l}n_{k}}$ can be built by considering the depth and number of descendants of each term in the DAG structure of each considered ontology (Acharya *et al.* 2017). To this aim, according to Teng *et al.* (2013), we computed the following measure of importance for each term *t* within the corresponding ontology, named structure-based Information Content (*ICstruct*):

(1) $$IC_{\mathrm{struct}}(t_{j,k})=\frac{\mathrm{depth}(t_{j,k})}{\max\_\mathrm{dept}h_{k}}\times \left(1-\frac{\left(\log(\mathrm{desc}(t_{j,k})+1\right)}{\log(\mathrm{total}\_\mathrm{term}s_{k})}\right)$$

where $t_{j,k}$ is the *j*th term in the *k*th ontology, $\mathrm{depth}(t_{j,k})$ and $\mathrm{desc}(t_{j,k})$ are the maximum depth and the number of descendants of the term $t_{j}$ in the ontology *k*, respectively, and $\max\_\mathrm{dept}h_{k}$ and $\mathrm{total}\_\mathrm{term}s_{k}$ are the maximum depth and the total number of terms of the ontology *k*, respectively. Division by $\max\_\mathrm{dept}h_{k}$ in (1) ensures that the $IC_{\mathrm{struct}}$ for each term in the ontology *k* falls in the range [0,1], allowing the $IC_{\mathrm{struct}}$ of multiple ontologies to be combined. Each entry in the weighted annotation matrix *W* is obtained by replacing the one-values of the binary annotation matrix with the *ICstruct* value of the corresponding term. The weighted annotation matrix *W* can then be used to define, for each gene, our proposed score of biological relevance called *Gene Information Score* (*GIS*), computed as:

(2) $$\mathrm{GIS}(g)=\frac{\sum_{m:W_{g,t_{m}}>0}W_{g,t_{m}}}{\sum_{m:W_{g,t_{m}}>0}1}$$

where $W_{g,t_{m}}$ is the weight for the term $t_{m}$ associated with the gene *g* in the matrix W. The above formula returns *GIS* values in the range [0,1], where a value of GIS(g)=0 indicates that no prior biological information exists for the gene *g* in the considered knowledge bases. The higher the *GIS* for a given gene, the higher the number of terms associated with it.

### 2.2 LASSO regularized models

Consider a n×q matrix *X*, where *n* is the number of samples in a dataset and *q* is the number of covariates, and the vector of observed outcomes $Y={\left(y_{i},\ldots y_{n}\right)}^{T}$ for the *n* samples. Assuming the outcome vector *Y* is generated from a specific distribution belonging to the canonical exponential family, as is usually the case, we can build a generalized linear model (GLM) to describe the relationship between *Y* and the data matrix *X*. The conditional distribution of $y_{i}$ given $x_{i}$ can be defined as:

(3) $$f(y_{i},z_{i}^{T}\beta )\propto \;\text{exp}[y_{i}z_{i}^{T}\beta -b(z_{i}^{T}\beta )]$$

where $z_{i}={\left(1,x_{i}^{T}\right)}^{T}$, $\beta ={\left(\beta_{1},\ldots ,\beta_{n}\right)}^{T}$ is the set of unknown parameters, and *b* is a log-partition function of a given distribution within the exponential family. A generalized linear model defines a canonical link function *m* that relates a linear model to the response variable *Y*, and this link function *m* can be written as:

(4) $$m={\left(b^{\prime }(z_{i}^{T}\beta )\right)}^{-1}$$

where $b^{\prime }$ is the first derivative of *b*. The loss function of a generalized linear model is defined as:

(5) $$L(\beta ;X,Y)=-\frac{1}{n}\sum_{i=1}^{n}[y_{i}z_{i}^{T}\beta -b(z_{i}^{T}\beta )]$$

and the set of parameter estimates $\hat{\beta }$ can be obtained by minimizing the loss function L(β;X,Y).

The LASSO regularization shrinks the model parameters by adding a $L_{1}$-norm penalty term to the loss function (Tibshirani 1996). The added term penalizes the sum of the absolute value of the parameters. Thus, a generalized linear model with LASSO regularization minimizes the following objective function:

(6) $$L_{\lambda }(\beta ;X,Y)=-\frac{1}{n}\sum_{i=1}^{n}[y_{i}z_{i}^{T}\beta -b(z_{i}^{T}\beta )]+\lambda \sum_{j=1}^{q}\left|\beta_{j}\right|$$

where λ represents the regularization parameter that controls the amount of shrinkage applied to the model parameters.

### 2.3 Weighted LASSO

The weighted LASSO regularization is an extension of LASSO regularization where the model parameters are penalized using different data-derived or prior-derived penalties. The weighted LASSO loss function can be derived from (6) by introducing for each model parameter $\beta_{j}$ a covariate-specific penalty $w_{j}$. A generalized linear model with weighted LASSO regularization minimizes the following objective function:

(7) $$L_{\lambda }(\beta ;X,Y)=-\frac{1}{n}\sum_{i=1}^{n}[y_{i}z_{i}^{T}\beta -b(z_{i}^{T}\beta )]+\lambda \sum_{j=1}^{q}w_{j}\left|\beta_{j}\right|$$

Thus, if all covariate-specific weights ${\left\{w_{j}\right\}}_{j=1}^{q}$ are set to 1, the weighted LASSO estimates degenerate to standard LASSO estimates. Each covariate-specific weight $w_{j}$ modifies the strength of the regularization applied to each parameter $\beta_{j}$: the larger the weight, the stronger the regularization, and the smaller the corresponding estimate, and vice versa.

In our approach, we propose to use prior known biological information, in the form of our proposed Gene Information Score, as individual penalties in a weighted LASSO model. Our goal is to penalize more the coefficient estimates of features/genes that have no prior biologically relevant information. Thus, the *GIS* cannot be directly incorporated into the optimization function of the weighted LASSO algorithm, as the higher the *GIS*, the higher the biological relevance of the corresponding feature. Therefore, we transform the computed *GIS* for each feature *j* through a parametrizable function as follows:

(8) $$w_{\mathrm{GIS}}(j)=\frac{1}{1+\mathrm{GIS}(j)}$$

As a result, in our *GIS*-weighted LASSO the penalty term λ is rescaled by a feature-specific penalty $w_{\mathrm{GIS}}$, which reduces the amount of regularization applied to features/genes that carry more biologically relevant information, as follows:

(9) $$L_{\lambda }(\beta ;X,Y)=-\frac{1}{n}\sum_{i=1}^{n}[y_{i}z_{i}^{T}\beta -b(z_{i}^{T}\beta )]+\lambda \sum_{j=1}^{q}w_{GIS_{j}}\left|\beta_{j}\right|$$

Indeed, the higher the *GIS*, the lower the corresponding $w_{\mathrm{GIS}}$ value. By construction, the *GIS* is always nonnegative and bounded at 1; thus, the corresponding $w_{\mathrm{GIS}}$ is nonnegative as well and bounded in the range [0.5,1].

The developed *GIS*-weighted LASSO regularized models are implemented in Python using the scikit-learn package (https://scikit-learn.org/stable/). The Python code for the computation of the *Gene Information Scores* and the implementation of our proposed approach is available at https://github.com/DEIB-GECO/GIS-weigthed_LASSO.

## 3 GIS sensitivity analyses

We conducted two distinct analytical studies to evaluate the impact of incorporating our proposed *Gene Information Score* into LASSO regularization.

The first study aimed at evaluating how the *GIS* score affects the feature selection process in the presence of multicollinearity. Multicollinearity refers to a statistical phenomenon in linear or logistic regression models where the predictor variables exhibit strong correlations with each other (Farrar and Glauber 1967). Such a phenomenon is rather common in large datasets. Indeed, multicollinearity is a considerable problem in gene-expression data analysis, as co-expressed genes often present high correlations. The presence of collinearity increases the variability of model parameter estimates, leading to inaccurate conclusions about the relationship between explanatory and response variables. Moreover, when a group of highly correlated features is present, LASSO has the tendency to select amongst them arbitrarily (Zou and Hastie 2005). By incorporating prior knowledge into the learning process, the model might be able to focus on one or only a few of the correlated features, being also those more biologically relevant, obtaining more robust and reliable parameter estimates. Thus, the goal of our first evaluation was to check whether, among features highly correlated, our proposed approach can identify and select only the one with the most known biological information.

The second study, instead, aimed at assessing the effect that the inclusion of prior biological knowledge has on the estimates of the model parameters. This analysis is of particular interest in scenarios where features with limited or no predictive power have high biological relevance and vice versa. The goal is to identify features with the best trade-off in terms of predictive power and biological relevance.

To perform such analyses, we built a controlled dataset with limited correlation among the features. We considered publicly available RNA-seq profiles of Kidney Renal Clear Cell Carcinoma patients from The Cancer Genome Atlas (TCGA) project (Weinstein *et al.* 2013). After preprocessing (Supplementary Section S1.1 for details), the dataset included 16 890 genes and 144 samples (72 healthy and 72 cancerous samples). Next, we performed an initial classification task with a *GIS*-weighted LASSO regularized logistic regression, aiming at distinguishing between cancer and noncancer samples in the dataset balanced by construction. We used an iterative procedure to initially select five features (Table 1) with different predictive values, in terms of Fisher’s score (Duda *et al.* 2001), and with Pearson’s correlations <0.5 with each of the other features in the original dataset. We then randomly extracted other 995 features, from the pool of the 16 885 left, having a correlation of <0.5 with the 5 selected features and with a correlation of <0.70 with each of the other features in the original dataset. The $w_{\mathrm{GIS}}$ scores used for this controlled dataset in the *GIS*-weighted LASSO regularized model were computed as described in Supplementary Section S2, considering only the Gene Ontology (GO) as the knowledge base of interest. The distributions of Pearson’s correlation values, Fisher’s scores, and $w_{\mathrm{GIS}}$ values in the controlled dataset are shown in Supplementary Fig. S1.

**Table 1.** Summary information of the five selected features/genes.

Feature/gene	Fisher’s score	$w_{GIS}$
*METTL8*	0.0	0.854
*PCBP1*	0.159	0.874
*NCKAP1*	0.317	0.845
*MT1F*	0.475	0.889
*PIK3C2G*	9.908	0.857

### 3.1 Multicollinearity analysis

To assess the effect of the proposed *GIS* on weighted LASSO regularization in the presence of multicollinearity, we performed the following experiments on the created controlled dataset. Separately for each of the selected five features in Table 1, in the dataset we generated 10 copies of the feature by adding Gaussian noise, with zero mean and 0.01 standard deviation, on the feature expression profile. Furthermore, we tested four different values for the regularization parameter λ (λ = [0.20, 0.25, 0.50, 1.0]), to evaluate how the strength of the regularization affects the results. For each selected feature/gene *g* and its 10 generated copies $g_{c}$, we performed the binary classification between cancer and noncancer samples using the *GIS*-weighted LASSO regularized logistic regression under the following five different *GIS* scenarios:

- NO *GIS*: Neither the selected original feature nor its added copies are assumed to have prior biological knowledge (standard LASSO), i.e. $w_{\mathrm{GIS}}(g)=1$ and $w_{\mathrm{GIS}}(g_{c})=1$
- *GIS* 1: The added copies are assumed to have no prior biological knowledge, i.e. $w_{\mathrm{GIS}}(g_{c})=1$
- *GIS* 2: The added copies are assumed to have little prior biological knowledge, with $w_{\mathrm{GIS}}(g_{c})$ values evenly spaced in the range [0.95,1.0]
- *GIS* 3: The added copies are assumed to have variable prior biological knowledge, with $w_{\mathrm{GIS}}(g_{c})$ values evenly spaced in the range [$w_{\mathrm{GIS}}(g)$,1.0]
- *GIS* 4: The added copies are assumed to have variable prior biological knowledge, with $w_{\mathrm{GIS}}(g_{c})$ values evenly spaced in the entire possible range [0.5,1.0].

If not specified otherwise, the $w_{\mathrm{GIS}}(g)$ of the original feature was left unchanged. This experiment was repeated 100 times for each feature, value of λ, and *GIS* scenario, and each time the classification model was fitted to the enlarged dataset with the 10 additional feature copies.

Out of the five selected features, genes *METTL8* and *PCBP1* were considered nonpredictive by both the standard and the *GIS*-weighted regularized models, given their original very low predictive value for the task. Their corresponding model coefficients were shrunk to zero and none of their generated copies were selected in any replica of the performed experiments. The gene *NCKAP1* or its copies (Supplementary Fig. S2) were never selected by the standard LASSO regularized model (NO *GIS*), due to its low predictive power. Instead, the *GIS*-weighted LASSO regularized model tended to select the *NCKAP1* gene for the *GIS* 1 and *GIS* 2 scenarios with λ=0.20, while some of its copies were selected for *GIS* 3 and *GIS* 4 scenarios. This is what is expected, as the amount of biological knowledge associated with the selected copies of the *NCKAP1* gene is on par or higher than the amount of the original feature itself. Furthermore, as a stronger regularization is applied, the *GIS*-weighted LASSO regularized model is able to discriminate between the multiple copies, and only select the one(s) with the highest biological relevance.

**Figure 1.**
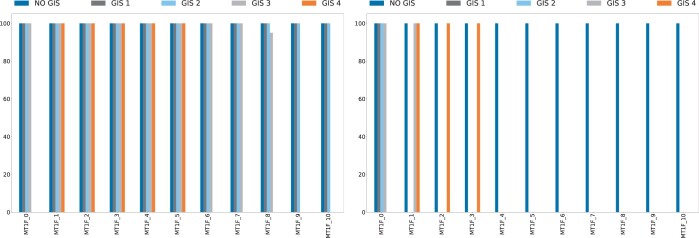
Multicollinearity analysis results for λ=0.20 and λ=1.0 for the *MT1F* gene across the different *GIS* scenarios described in Section 3.1. Each bar indicates how many times each feature, the original one or a copy of it, is selected across 100 repetitions. Bars are grouped by feature: in each diagram, the first set of bars (*MT1F*_0) on the left refers to the original feature, and the remaining 10 sets (*MT1F*_1, …, *MT1F*_10) refer to its copies.

Results for the experiments with λ=0.20 and λ=1.0 on the *MT1F* gene are shown in Fig. 1, while results for the *PIK3C2G* gene are reported in Supplementary Fig. S3; additional diagrams for the other considered values of the regularization parameter λ are shown in Supplementary Fig. S4. Results for *MT1F* show that the standard LASSO regularized model (NO *GIS*) always selected the original features and all its copies, regardless of the strength of the applied regularization (see also Supplementary Table S3). By increasing the regularization strength, the *GIS*-weighted LASSO regularized model is able to differentiate among the multiple copies of the gene, selecting only the original feature in the simpler *GIS* scenarios (*GIS* 1 and *GIS* 2) with stronger regularization. In the *GIS* 3 scenario with λ=1.0, both the original *MT1F* gene and one of its copies are selected, which by design are assigned the same $w_{\mathrm{GIS}}$ value. In the more complex scenario (*GIS* 4), with λ=1.0 the *GIS*-weighted LASSO regularized model selected three copies of the *MT1F* gene having the largest prior biological knowledge. Similar results are obtained for the *PIK3C2G* gene. Yet, due to its high predictive power (see Table 1), the original feature and/or multiple copies are selected by the *GIS*-weighted LASSO regularized model, even when stronger regularization is applied.

These results show that the proposed *GIS*-weighted LASSO regularization does not overturn the main outcomes of standard feature selection. Yet, our proposal is able to better module the selection of predictive correlated features based on their prior biological knowledge.

### 3.2 Predictive power analysis

We assessed the effect of including prior biological knowledge on the selection of features, also simulating scenarios in which the prior knowledge associated with each feature is incomplete (higher $w_{\mathrm{GIS}}$) or present some errors (potentially lower or higher $w_{\mathrm{GIS}}$). Findings from this analysis and related discussion are in Supplementary Section S3.3. Results show that the incorporation of prior knowledge through feature-specific penalties modifies the estimated model coefficients. Indeed, smaller $w_{\mathrm{GIS}}$ values correspond to larger estimates of the coefficients as less regularization is applied. Nonetheless, each feature’s predictive power still controls its selection, as features with limited or no predictive power were never selected with the *GIS*-weighted LASSO regularization, even for small feature-specific penalties.

## 4 Application use cases

In this section, we illustrate and compare the performance of the standard regularized LASSO model and our proposed approach on two application use cases concerning the cancer-related subtype prediction of patients based on gene expression data. The $w_{\mathrm{GIS}}$ scores used in the *GIS*-weighted regularization are computed as described in Supplementary Section S2, considering multiple knowledge bases of interest. In addition, we present findings from Supplementary experiments that compare and assess the performance of our proposed approach against several widely used feature selection methods in gene expression analysis on the two considered application use cases.

### 4.1 Breast cancer subtyping

The first application use case concerns the classification of Breast Invasive Carcinoma patients in their corresponding cancer subtypes. The used dataset (Weinstein *et al.* 2013, Pallotta *et al.* 2022) and the applied pre-processing steps are described in Supplementary Section S1.2. The developed regularized multi-class logistic regression classifiers were trained according to the procedure described in Supplementary Section S4. Classification results are reported in Table 2. Additional information on the performance of the classifiers is reported in Supplementary Table S5. The standard and the *GIS*-weighted LASSO regularized models achieved similar performance in cross-validation and on the test set, with the latter model selecting a higher number of features (+10.3%).

**Table 2.** Classification performance (mean ± standard deviation) and number of features selected as union or intersection across 10 training set sample permutations.^a^

	Breast cancer				Colorectal cancer			
Regularization	CV accuracy	Test accuracy	Union	Intersection	CV accuracy	Test accuracy	Union	Intersection
LASSO	0.90427 (±0.0)	0.93564 (±0.0)	195	194	0.76665 (±0.00093)	0.83871 (±0.0)	371	369
$w_{\mathrm{GIS}}$	0.91047 (±0.0)	0.93069 (±0.0)	214	214	0.77210 (±0.0)	0.83871 (±0.0)	420	420

a CV: cross-validation. $w_{\mathrm{GIS}}$ refers to GIS-weighted LASSO, with penalty as in Equation (8).

Performed biological validation analyses of the selected features are described in Supplementary Section S5; their results are summarized in Table 3 and Supplementary Table S7. The standard and *GIS*-weighted LASSO regularized models share a relevant percentage of predictive genes for each BRCA subtype of interest ($N_{shared}$, Table 3), indicating that incorporating prior information does not affect the ability of the classifier to select the most predictive genes in terms of their discriminative power. In addition, the *GIS*-weighted LASSO regularization is able to favor the selection of more biologically informative genes when features contribute equally to the predictive task. The number of distinct genes, across the four BRCA subtypes, selected only by the standard LASSO regularization, was 18, whereas the corresponding number for the *GIS*-weighted LASSO regularization was 39. Indeed, the latter ones have a greater number of unique GO, Reactome biological pathway, and HPO annotation terms, and a percentage of these terms enriched, for each different BRCA subtype. Only five DisGeNET BRCA-specific gene signatures included at least one of the genes selected only by the standard LASSO regularization, with *Breast Carcinoma* and *Malignant Neoplasm of Breast* as the most represented signatures (with 5 of the 18 selected genes). Conversely, 20 different DisGeNET BRCA-specific gene signatures included at least one of the *GIS*-weighted LASSO selected genes, with 18 and 17 of the 39 selected genes included in the *Breast Carcinoma* and *Malignant Neoplasm of Breast* signatures, respectively. Furthermore, only 2 of the 18 genes selected only by the standard LASSO were included in the Malacards BRCA-specific gene signature (of 1034 genes), whereas 10 of the 39 genes selected only by the *GIS*-weighted LASSO were found in such a signature. We also found that 5 and 3 genes selected only by the *GIS*-weighted LASSO belong to cancer-specific or BRCA-specific KEGG pathways, respectively. Instead, no overlap was found between these KEGG pathway signatures and the genes selected only by the standard LASSO regularization.

**Table 3.** Enrichment analysis results for BRCA subtyping.^a^

Subtype	N${}_{\mathrm{LASSO}}$	N${}_{w_{\mathrm{GIS}}}$	N${}_{shared}$	GO${}_{\mathrm{LASSO}}$	GO${}_{w_{\mathrm{GIS}}}$	Reactome${}_{\mathrm{LASSO}}$	Reactome${}_{w_{\mathrm{GIS}}}$	HPO${}_{\mathrm{LASSO}}$	HPO${}_{w_{\mathrm{GIS}}}$
Basal	30	30	25	1564 (8.0%)	1871 [1.20] (16.0%^b^)	104 (0%)	105 [1.01] (0%)	967 (0%)	1182 [1.22] (0%)
Her2	51	56	46	2376 (0%)	2685 [1.13] (1.4%^b^)	361 (0.8%)	381 [1.06] (0.8%)	921 (0%)	1346 [1.46] (0.2%^b^)
LumA	76	86	71	2747 (6.2%)	3159 [1.15] (7.7%^b^)	346 (12.7%)	495 [1.43] (12.5%)	1519 (1.1%)	1980 [1.30] (3.7%)
LumB	57	63	53	2267 (1.3%)	2518 [1.11] (1.2%)	164 (5.5%)	245 [1.49] (3.7%)	1165 (7.9%)	1602 [1.38] (9.2%)

a Number of GO, reactome biological pathway, or HPO annotation terms retrieved for each selected feature subset of size *N* (and percentage of significantly enriched terms, with FDR *P*-value < 0.05). $w_{\mathrm{GIS}}$ refers to *GIS*-weighted LASSO, with penalty as in Equation (8). [·]: ratio between the number of retrieved terms annotated to the gene subsets selected by the *GIS*-weighted and standard LASSO regularization.

b Statistically significant percentage of enriched terms with respect to the standard LASSO-related ones (*Z*-test for proportions, *P*-value < 0.05).

### 4.2 Colorectal cancer subtyping

The second application use case, instead, focuses on the classification of Colorectal cancer patients in their corresponding cancer subtypes. Details about the used dataset (Weinstein *et al.* 2013, Pallotta *et al.* 2022) and the applied preprocessing steps can be found in Supplementary Section S1.3. The developed regularized multi-class logistic regression classifiers were trained following the procedure outlined in Supplementary Section S4. The results of the classification are presented in Table 2. Further information about the performance of the classifiers is reported in Supplementary Table S6. The standard and the *GIS*-weighted LASSO regularized models reached comparable performances in cross-validation and on the test set. Yet, the *GIS*-weighted LASSO regularization selected a higher number of features than the corresponding standard LASSO (+13.8%). Biological validation of selected classification features/genes was performed as described in Supplementary Section S5; the obtained results are reported in Table 4 and Supplementary Table S8. As for the previous application use case, the standard and *GIS*-weighted LASSO regularized models share a significant percentage of selected features for each distinct CRC subtype ($N_{shared}$, Table 4). This further shows that integrating prior information does not influence the classifier’s ability to identify the most discriminative genes.

**Table 4.** Enrichment analysis results for CRC subtyping.^a^

Subtype	N${}_{\mathrm{LASSO}}$	N${}_{w_{\mathrm{GIS}}}$	N${}_{\mathrm{shared}}$	GO${}_{\mathrm{LASSO}}$	GO${}_{w_{\mathrm{GIS}}}$	Reactome${}_{\mathrm{LASSO}}$	Reactome${}_{w_{\mathrm{GIS}}}$	HPO${}_{\mathrm{LASSO}}$	HPO${}_{w_{\mathrm{GIS}}}$
CRIS A	67	72	63	2304 (0.7%)	2520 [1.10] (1.6%^b^)	219 (1.4%)	253 [1.16] (1.2%)	1054 (0.1%)	1542 [1.46] (0.1%)
CRIS B	81	94	68	2067 (0.1%)	2781 [1.35] (0.1%)	266 (0.4%)	406 [1.53] (0.5%)	1379 (0%)	2384 [1.73] (0.1%)
CRIS C	74	81	64	2494 (0.3%)	2789 [1.12] (1.4%^b^)	365 (0%)	409 [1.12] (1.0%^b^)	1156 (0%)	1590 [1.38] (0%)
CRIS D	92	104	86	3029 (0%)	3364 [1.11] (0%)	302 (0%)	377 [1.25] (0%)	1994 (0.1%)	2413 [1.21] (0%)
CRIS E	86	96	80	2416 (0%)	2853 [1.18] (0%)	194 (0%)	222 [1.14] (0%)	1609 (0.1%)	1842 [1.15] (0.1%)

a Number of GO, reactome biological pathway, or HPO annotation terms retrieved for each selected feature subset of size *N* (and percentage of significantly enriched terms, with FDR *P*-value < 0.05). $w_{\mathrm{GIS}}$ refers to *GIS*-weighted LASSO, with penalty as in Equation (8). [·]: ratio between the number of retrieved terms annotated to the gene subsets selected by the *GIS*-weighted and standard LASSO regularization.

b Statistically significant percentage of enriched terms with respect to the standard LASSO-related ones (*Z*-test for proportions, *P*-value < 0.05).

On the other hand, indeed it allows the selection of more informative genes, for a better biological interpretation of the results. A total of 38 genes, across the five distinct CRC subtypes, were selected only by the standard LASSO regularization, whereas the corresponding number of genes selected only by the *GIS*-weighted LASSO regularization was 86; the latter ones have relevantly more GO, Reactome biological pathway, and HPO annotation terms, and percentage of them enriched, for each CRC subtype. The number of distinct DisGeNET CRC-specific gene signatures including at least one of these genes from the standard or *GIS*-weighted LASSO regularization was 3 and 7, respectively. The most represented DisGeNET CRC-specific signatures were *Colorectal Carcinoma* and *Colorectal Cancer*, both with 6 of the 38 genes selected only by the standard LASSO regularization, and with 27 and 28 of the 86 genes only from the *GIS*-weighted LASSO regularization, respectively. Furthermore, the Malacards CRC-specific gene signature (of 921 genes) included only 2 of the 38 genes selected only by the standard LASSO regularization versus 14 of the 86 genes selected only by the *GIS*-weighted LASSO regularization (*Z*-test for proportions, *P*-value < 0.05). Finally, we found no overlap between the distinct genes selected only by the standard LASSO regularization and those in the KEGG cancer-specific or CRC-specific pathway gene signatures. Instead, we identified nine genes associated with KEGG cancer-specific pathways and one gene linked to KEGG CRC-specific pathways among the genes selected only by the *GIS*-weighted LASSO regularization.

### 4.3 Comparative performance evaluation

We performed several experiments to assess the performance of the proposed *GIS*-weighted LASSO regularized model in comparison to various other feature selection methods on the BRCA and CRC datasets. Given that LASSO is an embedded feature selection method, we included both filter- and wrapper-based methods in our analysis. For the filtering methods, we evaluated Mutual Information (MI) (Shannon 1948, Battiti 1994), Fisher’s scores (FI) (Duda *et al.* 2001), minimum Redundancy-Maximum Relevance (mRMR) (Ding and Peng 2003), Kolmogorov-Smirnov (KS) (Biesiada and Duch 2007), and the Relief algorithm (Kira and Rendell 1992). As for the wrapper-based methods, we focused exclusively on forward feature selection (FS), as it offers more reliable estimates by incrementally adding features, thus enabling complete model fitting at each step. Further details on the experiments and the related results are available in Supplementary Section S6.1. Overall, embedded methods outperformed filter- and wrapper-based techniques. The performance of filter-based methods varied depending on the feature selection algorithm and dataset, whereas the *GIS*-weighted LASSO appeared to be less sensitive to the dataset being analyzed, achieving competitive classification performances while steering the selection toward more biologically relevant genes, in terms of existing available knowledge. In addition, we examined whether using an integrated approach to incorporate prior information would yield improved results compared to using a traditional filter-based prior knowledge approach. We used the proposed *Gene Information Score* (GIS) as prior biological knowledge. As a baseline, we selected feature subsets comparable in size to the ones obtained with the embedded approaches, by considering only each gene’s prior information. In addition, we applied two filtering strategies that considered both prior biological knowledge and discriminative power. In one strategy, we initially selected genes based on their discriminative power (Fisher’s scores), followed by selecting genes based on their biological relevance. Alternatively, in the second strategy, we first filtered genes based on their biological relevance and then refined the selection by their discriminative power. Further details on these experiments and their results are available in Supplementary Section S6.2. While such approaches appeared to retrieve more significant annotations and disease-specific genes, they all fell short in classification accuracy compared to our proposed integrated approach. In contrast, our method excelled in both classification and selection of biologically relevant features, allowing for improved classification performance and biological interpretability.

## 5 Discussion

In this work, we introduced an embedded integrative method for feature selection that combines weighted LASSO feature selection and prior biological knowledge in a single step. We proposed a flexible framework that integrates and combines multiple sources of prior knowledge on gene functions and properties to enhance the biological interpretability of the results. Our approach is based upon a novel score of biological relevance, called *Gene Information Score*, that summarizes the prior existing information for each gene across one or more different biological knowledge bases. Indeed, the goal of our proposal is to find a trade-off between the predictive power of each selected gene, with respect to the prediction task, and its biological relevance. We showed, through distinct sensitivity analyses on a controlled dataset, that incorporating prior knowledge in gene-expression data analysis: (i) can alleviate the effect of multicollinearity, and (ii) does not heavily affect the estimates of the model parameters, guaranteeing the selection of the most predictive features.

In the real data examples, based on two distinct use case applications of biological and clinical relevance, we demonstrated the effectiveness of our proposed *GIS*-weighted LASSO approach compared to the standard LASSO regularization and other popular feature selection algorithms for gene expression data analysis. Results show that incorporating prior information does not affect the ability of the classifier to select the most predictive genes based on their discriminative power. The *GIS*-weighted LASSO regularization is able to identify the most relevant genes while simultaneously enhancing the biological interpretability that the selected features can provide, as demonstrated by the biological validation analyses we conducted. Indeed, our approach closely aligns with the concept of interpretability. Its primary goal is not just to identify predictive genes, but also to potentially understand their underlying biological roles in generating the observed phenotypes and improve our comprehension of the biological mechanisms involved.

By introducing a score of biological relevance that can be directly incorporated into the learning process of a LASSO regularized algorithm, we provide a flexible and general strategy for prior-knowledge-guided feature selection approaches. As biological knowledge is progressively collected and accumulated over time from experimental studies and analyses, additional information can be considered in the calculation of the *Gene Information Score*, potentially further enhancing the interpretability of the results as well as improving the prediction performance. Our approach can consider all existing information stored in the knowledge bases of interest, providing a general and complete picture of each gene’s biological relevance. In this way, it does not require users to provide domain-specific external information, but only to determine the knowledge bases to be used in the *Gene Information Score* calculation. Nonetheless, if domain-specific information is available, our proposed score can easily include only knowledge base terms associated with the specific information of interest.

Inspired by Bergersen *et al.* (2011), we used a parametrizable function to calculate feature-specific penalties. Different hyperparameters can be defined to control and determine the shape of the function that computes the $w_{\mathrm{GIS}}$ scores. By modifying the shape of the function, the relative strength of the $w_{\mathrm{GIS}}$ weights can be set, i.e. it is possible to control how much we want to consider and prioritize/penalize genes with high/low prior biological relevance. These hyperparameters can be easily tuned together with the regularization parameter λ using *k*-fold cross-validation and a grid-search approach.

By design, our *Gene Information Score* enables the integration of multiple knowledge bases, based on the assumption that they can be represented as an ontology, with a controlled vocabulary of terms related to each other. Nonetheless, biological databases that do not have a hierarchical organization and are structured as plain terminologies might also be considered in the computation of the score. Moreover, our score can also accommodate graphs, considering their weighted adjacency matrices in the calculation. These require ad-hoc modifications of the formula in Equation (2) to account for differences in the structure of the integrated sources, which is left for future work. It should also be noted that the considered prior biological knowledge may be incomplete or present errors. While our approach does not resolve this general issue, the proposed biological relevance score can be readily extended to also incorporate information on the reliability and correctness of the prior knowledge, if available, potentially limiting the effect of incomplete or erroneous information.

We limited our analysis to the study of logistic regression with LASSO regularization, but several alternatives and generalizations are possible, including extensions to all generalizable linear models. Future work will focus on the extension of our approach to other penalty terms, such as Ridge and Elastic-Net penalties. Furthermore, extensions to other nonlinear regression/classification algorithms are possible and will be explored, including tree-based models. In this work, we considered application use cases concerning the cancer-related subtype prediction of patients based on gene expression data. Nonetheless, our proposed approach is quite versatile and applicable to datasets in bioinformatics or any other related or unrelated field.

## Supplementary Material

btae605_Supplementary_Data

## Data Availability

The code and the controlled dataset are available at https://github.com/DEIB-GECO/GIS-weighted_LASSO. The BRCA and CRC datasets can be downloaded from https://www.cbioportal.org.
