# Supplementary material for: Biologically weighted LASSO: enhancing functional interpretability in gene expression data analysis
Source: Bioinformatics. 2024 Oct 16;40(10):btae605. doi: 10.1093/bioinformatics/btae605 (PMC11639179; doi:10.1093/bioinformatics/btae605)
Supplement: btae605_Supplementary_Data [file btae605_supplementary_data.pdf]

Supplementary material
Biologically weighted LASSO: enhancing functional interpretability in gene expression data analysis

Sofia Mongardi, Silvia Cascianelli, Marco Masseroli

Dipartimento di Elettronica, Informazione e Bioingegneria (DEIB), Politecnico di Milano, Via Ponzio 34/5, 20133, Milan, Italy

S1 Materials and Methods

S1.1 Kidney Renal Clear Cell Carcinoma Dataset

We created a gene expression dataset including publicly available RNA-seq profiles of Kidney Renal Clear Cell Carcinoma patients from The Cancer Genome Atlas (TCGA) [1]. The original dataset included gene expression data for 60,483 genes and 611 samples. The gene expression profiles were pre-processed as follows. We first discarded miRNA genes (1,751), then removed the samples with the top-five expressed genes accounting for at least 20% of the total sample raw counts (greedy samples), and lastly eliminated the genes (29,617) that were not expressed in at least 80% of the samples. We considered as not expressed the genes with raw counts ≤ 4. We also removed all the non-coding genes (according to the NCBI Reference Sequence Database annotation [2]). Next, we normalized the gene raw counts by computing the *reads per million* (RPM) of each gene g_j in each sample s_i as: $RPM = \frac{\#\ reads\ mapped\ to\ g_j}{total\ \#\ reads\ for\ sample\ s_i} * 10^6$, and then a log_2 transformation of the RPM values. Because the dataset was highly imbalanced (72 healthy samples and 539 cancerous samples), we constructed a balanced dataset by randomly sampling 72 of the cancerous patients. The balanced final dataset had a total of 144 samples and 16,890 genes.

S1.2 Breast Cancer Subtyping Dataset

This dataset included publicly available RNA-seq profiles of Breast Invasive Carcinoma patients from TCGA [1]. The original dataset included gene expression data of 25,150 genes and 1,053 samples. We pre-processed this gene expression data as described in Section S1.1. We discarded miRNA genes (1,650), removed the greedy samples (10), discarded the not expressed genes (1,421), and then retained only the coding genes (17,924). We normalized the gene raw counts by computing the *reads per million* (RPM). We used this pre-processed gene-expression data to classify their breast cancer (BRCA) patient samples into the corresponding intrinsic subtypes. We retrieved the BRCA intrinsic subtypes for the pre-processed TCGA samples (1,043) from cBioPortal[1], obtained from the state-of-the-art PAM50 test [3]. Our analyses focused on the Basal (175), HER2-enriched (Her2, 81), Luminal A (LumA, 543), and Luminal B (LumB, 207) subtypes, totaling 1,006 patients/samples (see Table S1). The Normal-like class (37) was excluded from our study due to its controversial clinical significance.

[1] `https://www.cbioportal.org/`

Table S1: Class distributions in the training and testing sets for the Breast Cancer and Colorectal Cancer datasets.

Dataset	Breast Cancer					Colorectal Cancer					
Set	Basal	Her2	LumA	LumB	Total	CRIS A	CRIS B	CRIS C	CRIS D	CRIS E	Total
Training	140	65	434	165	804	128	67	132	78	91	496
Test	35	16	109	42	202	32	16	33	20	23	124
All	175	81	543	207	1,006	160	83	165	98	114	620

S1.3 Colorectal Cancer Subtyping Dataset

This dataset included publicly available raw RNA-seq profiles of Colon Adenocarcinoma (COAD) and Rectum Adenocarcinoma (READ) patients from TCGA [1]. The original dataset included gene expression data of 58,387 genes and 620 primary tumoral samples. We pre-processed the gene expression data as described in Section S1.1. We first discarded miRNA genes (1,732), removed the greedy samples (0), discarded the not expressed genes (21,659), and then retained only the coding genes (17,559). We normalized the gene raw counts by computing the *reads per million* (RPM). After pre-processing, we used these gene-expression profiles to classify their colorectal cancer (CRC) patient samples into the corresponding ColoRectal Intrinsic Subtypes (CRIS), obtained through the Nearest Template Prediction (NTP) algorithm [4]. All the subtypes were considered in our analysis: CRIS A (160), CRIS B (83), CRIS C (165), CRIS D (98), and CRIS E (114), for a total of 620 samples (see Table S1).

S1.4 Regularized Models

Consider a $n \times q$ matrix X, where n is the number of samples in a dataset and q is the number of covariates, and the vector of observed outcomes $Y = (y_i, \cdots y_n)^T$ for the n samples. A generalized linear model with LASSO regularization minimizes the following objective function:

$$L_\lambda(\beta; X, Y) = -\frac{1}{n}\sum_{i=1}^{n}[y_i z_i^T \beta - b(z_i^T \beta)] + \lambda \sum_{j=1}^{q} |\beta_j| \quad (1)$$

where $z_i = (1, x_i^T)^T$, $\beta = (\beta_1, \cdots, \beta_n)^T$ is the set of unknown parameters, b is a log-partition function of a given distribution within the exponential family, and λ represents the regularization parameter that controls the amount of shrinkage applied to the model parameters.

S1.4.1 Linear Regression

Linear regression is a generalized linear model where the response variable y_i is assumed to be normally distributed, and the link function m is the identity (see Section *LASSO Regularized Models* in the main article). Consider a standard linear regression model $y_i = z_i^T \beta + \epsilon_i$, where y_i is the observed outcome for i^{th} sample, $z_i = (1, x_i^T)^T$, x_i is the corresponding gene expression profile, and ϵ_i is the error term sampled from a normal distribution with zero mean and variance σ^2. The loss function of the LASSO regularized linear regression is obtained from (1) with $b(z_i^T \beta) = (z_i^T \beta)^2/2$, and can be written as:

$$L_\lambda(\beta; X, Y) = -\frac{1}{n}\sum_{i=1}^{n}(y_i - z_i^T \beta)^2 + \lambda \sum_{j=1}^{q} |\beta_j| \quad (2)$$

S1.4.2 Logistic Regression

Logistic regression is a generalized linear model with a logit link function $m(p) = \ln(\frac{p}{1-p})$, and with the response variable Y from a Bernoulli distribution, therefore taking a value of 1 or 0. Instead

of modelling y_i directly, logistic regression models the probability $p(x_i) = p(y_i = 1|x_i)$ for each observation i. The probability $p(x_i)$ is computed using the logistic function as:

$$p(x_i) = \frac{\exp(z_i^T \beta)}{1 + \exp(z_i^T \beta)} \quad (3)$$

Setting $b(z_i^T \beta) = log(1 + exp(z_i^T \beta))$ in (1), the objective function of the LASSO regularized logistic regression can be defined as:

$$L_\lambda(\beta; X, Y) = -\frac{1}{n} \sum_{i=1}^{n} y_i log(p(x_i)) + (1 - y_i) log(1 - p(x_i)) + \lambda \sum_{j=1}^{q} |\beta_j| \quad (4)$$

S1.4.3 Multi-class Logistic Regression

Multi-class logistic regression is an extension of logistic regression to solve problems where the response variable Y has a multinomial distribution with C possible categories. Thus, the loss function of the LASSO regularized multi-class logistic regression is:

$$L_\lambda(\beta; X, Y) = -\frac{1}{n} \sum_{i=1}^{n} \sum_{c=1}^{C} \mathbf{1}[y_i = c] log(p_c(x_i)) + \lambda \sum_{j=1}^{q} |\beta_j| \quad (5)$$

where $\mathbf{1}[y_i = c]$ is an indicator function that returns 1 if $y_i = c$, or 0 otherwise, and $p_c(x_i)$ is the class probability for the cth category, computed as:

$$p_c(x_i) = \frac{\exp(z_i^T \beta_c)}{\sum_{l=1}^{C} \exp(z_i^T \beta_l)} \quad (6)$$

S2 Gene Information Score Calculation

To compute the proposed *Gene Information Score* (*GIS*), we considered the Gene Ontology (GO) [5], Reactome [6], and Human Phenotype Ontology (HPO) [7] as biological knowledge sources. The Gene Ontology is a standardized vocabulary of terms structured as a directed acyclic graph (DAG), representing term relationships. This ontology is composed of three sub-ontologies describing biological processes (BP), cellular components (CC), and molecular functions (MF). In the *GIS* computation, performed as described in the main article Section Materials and Methods, we considered the three subontologies as independent knowledge bases. Reactome is a curated biological pathway database with a hierarchical structure, making it possible to explore the details of specific pathways and infer their relationships. The Human Phenotype Ontology is a controlled vocabulary for describing phenotypic abnormalities associated with human diseases. Similarly to GO, HPO has a hierarchically organized ontology structure, where terms are linked to each other based on their relationships within a DAG. For each dataset under study, we retrieved all its gene annotations from each different knowledge base, associated with the dataset genes remaining after the pre-processing steps. All available GO annotation terms were retrieved using the biomaRt[2] R library (version 2.56.1). Particularly, for each gene only the most specific GO annotation terms (i.e., those at the highest depth in the DAG hierarchy, according to the true-path-rule [5]) are provided by biomaRt. Therefore, to obtain all the GO annotation terms associated with the dataset genes, we extracted all the ancestors at any depth level of the GO DAG (format-version 1.2, release date: 2023-03-06) of these specific GO terms, considering all and only the DAG *'is_a'* and *'part_of'* relationships. All Reactome pathway (version V85) and HPO (format-version 1.2, release date: 2023-09-01) annotation terms were obtained directly from each knowledge base website by downloading the relevant files. As for GO, we performed the unfolding of the specific Reactome pathway and HPO annotations by retrieving all the ancestors at any depth level of the corresponding DAG. We considered the *'is_a'* relationship for HPO, while we extracted the parent-child relationships for Reactome from the relevant file. A summary of the content of the different knowledge bases used is reported in Table S2.

[2] `https://bioconductor.org/packages/release/bioc/html/biomaRt.html`

Table S2: Summary information of the different knowledge bases used in the computation of the *Gene Information Score*.

Knowledge Base	GO: BP	GO: CC	GO: MF	Reactome	HPO
N term	30,540	12,471	4,473	2,629	17,895
Max depth	19	13	15	12	18

N term is the number of terms in the corresponding knowledge base.
Max depth is the maximum depth of the corresponding knowledge base.

S3 *GIS* Sensitivity Analyses

To assess the effect of using our proposed *GIS* in the LASSO regularized model, we created a controlled dataset and performed two different analytical studies on it. The goal of the first study was to evaluate the impact of the *GIS* on the feature selection process when dealing with multicollinearity. The second study, instead, focused on assessing how the incorporation of prior biological knowledge influences the estimates of parameters in prediction models.

S3.1 Controlled Dataset

Starting from the Kidney Renal Clear Cell Carcinoma, we built a controlled dataset as described in the main article Section *GIS* Sensitivity Analyses. Here, in Figure S1, we present the distributions of Pearson's correlation values, Fisher's scores, and w_{GIS} values in the controlled dataset.

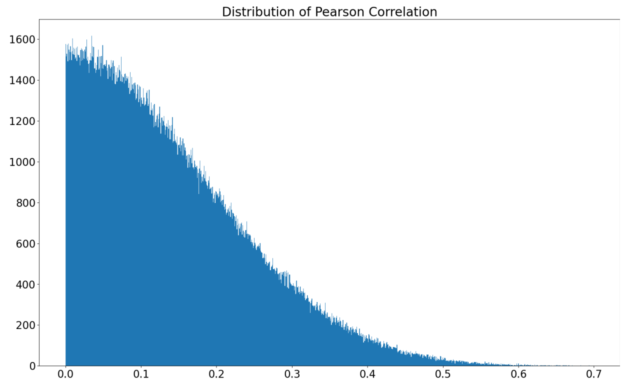

(a) Pearson's Correlation

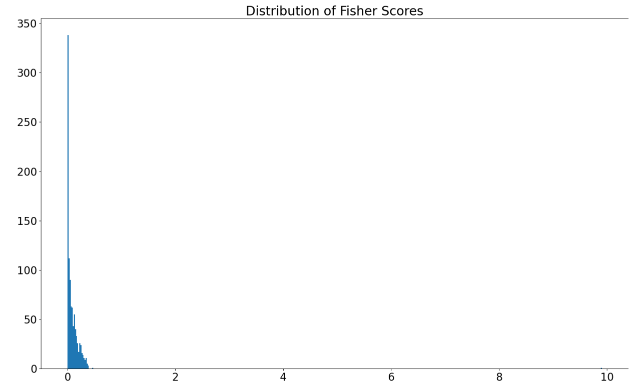

(b) Fisher's Scores

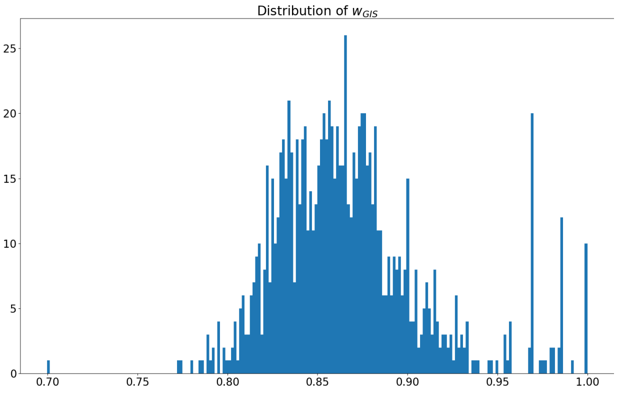

(c) w_{GIS}

Figure S1: Distributions of Pearson's correlation values (a), Fisher's scores (b), and w_{GIS} values (c) in the controlled dataset.

S3.2 Multicollinearity Analysis

In this section, we present additional results from the multicollinearity analysis within Table S3 and in Figures S2, S3, and S4.

Table S3: Summary results of the multicollinearity analysis.

		NCKAP1		*MT1F*		*PIK3C2G*	
GIS	λ	N_f	$Mean_{copies}$	N_f	$Mean_{copies}$	N_f	$Mean_{copies}$
NO *GIS*	0.20	0	0 (±0.0)	100	10 (±0.0)	100	10 (±0.0)
GIS 1	0.20	100	0 (±0.0)	100	10 (±0.0)	100	10 (±0.0)
GIS 2	0.20	100	0 (±0.0)	100	10 (±0.0)	100	10 (±0.0)
GIS 3	0.20	100	0.93 (±0.26)	100	7.95 (±0.22)	100	10 (±0.0)
GIS 4	0.20	0	4 (±0.0)	0	5 (±0.0)	100	8 (±0.0)
NO *GIS*	0.25	0	0 (±0.0)	100	10 (±0.0)	100	10 (±0.0)
GIS 1	0.25	0	0 (±0.0)	100	10 (±0.0)	100	10 (±0.0)
GIS 2	0.25	0	0 (±0.0)	100	8.77 (±0.47)	100	10 (±0.0)
GIS 3	0.25	0	0 (±0.0)	100	6.99 (±0.10)	100	10 (±0.0)
GIS 4	0.25	0	3.12 (±0.32)	0	5 (±0.0)	100	7 (±0.0)
NO *GIS*	0.50	0	0 (±0.0)	100	10 (±0.0)	100	10 (±0.0)
GIS 1	0.50	0	0 (±0.0)	100	0 (±0.0)	100	10 (±0.0)
GIS 2	0.50	0	0 (±0.0)	100	2.96 (±0.28)	100	9.97 (±0.17)
GIS 3	0.50	0	0 (±0.0)	100	3.98 (±0.14)	100	8.02 (±0.14)
GIS 4	0.50	0	2 (±0.0)	0	4 (±0.0)	0	5 (±0.0)
NO *GIS*	1.0	0	0 (±0.0)	100	10 (±0.0)	100	10 (±0.0)
GIS 1	1.0	0	0 (±0.0)	100	0 (±0.0)	100	7.17 (±0.81)
GIS 2	1.0	0	0 (±0.0)	100	0 (±0.0)	100	5.14 (±0.35)
GIS 3	1.0	0	0 (±0.0)	100	1 (±0.0)	100	4.99 (±0.10)
GIS 4	1.0	0	1.01 (±0.10)	0	3 (±0.0)	0	3.32 (±0.47)

N_f: number of times the original feature is selected in the 100 rounds.
$Mean_{copies}$: mean (± standard deviation) of the number of copies selected over the 100 rounds.
NO *GIS*: neither the selected original feature nor its added copies are assumed to have prior biological knowledge (standard LASSO), i.e., $w_{GIS}(g) = 1$ and $w_{GIS}(g_c) = 1$.
GIS 1: the added copies are assumed to have no prior biological knowledge, i.e., $w_{GIS}(g_c) = 1$.
GIS 2: the added copies are assumed to have little prior biological knowledge, with $w_{GIS}(g_c)$ values evenly spaced in the range [0.95,1.0].
GIS 3: the added copies are assumed to have variable prior biological knowledge, with $w_{GIS}(g_c)$ values evenly spaced in the range [$w_{GIS}(g)$,1.0].
GIS 4: the added copies are assumed to have variable prior biological knowledge, with $w_{GIS}(g_c)$ values evenly spaced in the entire possible range [0.5,1.0].

S3.3 Predictive Power Analysis

We performed the following experiments to evaluate the effect of the *GIS* on the model coefficients estimated during training of a *GIS*-weighted LASSO regularized model. As for the multicollinearity analysis, we performed the binary classification between cancer and non-cancer samples using the *GIS*-weighted LASSO regularized logistic regression and the controlled dataset described in Section S3.1. Separately for each of the five features considered, we trained multiple *GIS*-weighted LASSO regularized models, varying the gene w_{GIS} value each time. A total of 50 models were trained for each feature, employing 50 different w_{GIS} values equally spaced in the entire range [0.5,1.0]. As for the multicollinearity analysis, we tested four different values for the regularization parameter λ (λ = [0.20, 0.25, 0.50, 1.0]). Results from the experiments are shown in Figure S5. By increasing the strength of the applied regularization, differences in model coefficients as the corresponding feature w_{GIS} varies are more evident. Indeed, the lower the w_{GIS}, the higher the amount of prior biological knowledge of the corresponding gene, and the higher its corresponding model coefficient, as less reg-

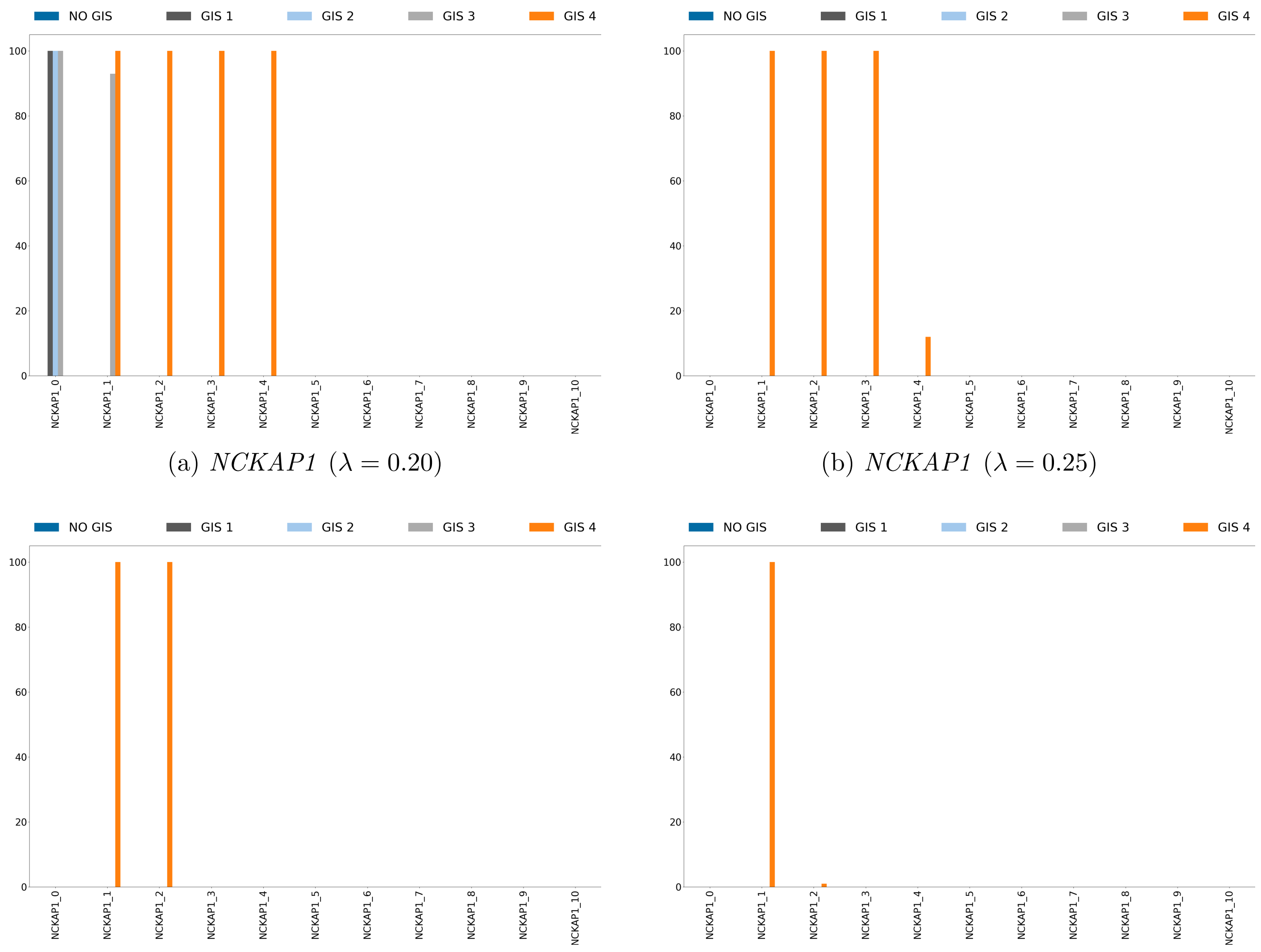

(a) *NCKAP1* ($\lambda = 0.20$)

(b) *NCKAP1* ($\lambda = 0.25$)

(c) *NCKAP1* ($\lambda = 0.50$)

(d) *NCKAP1* ($\lambda = 1.0$)

Figure S2: Multicollinearity analysis results for gene *NCKAP1*. Each bar indicates how many times each feature, the original one or a copy of it, is selected across 100 repetitions. Bars are grouped by feature: In each diagram, the first set of bars on the left refers to the original feature, and the other remaining 10 sets refer to its copies.

ularization is applied to it. Genes *METTL8* and *PCBP1*, even for small values of w_{GIS}, were never selected by the model, indicating that each feature's predictive power still controls its selection. As expected, the incorporation of prior knowledge through feature-specific penalties slightly affects the estimates of model coefficients. Nonetheless, the proposed *GIS*-weighted LASSO regularization does not significantly change the main outcomes of standard feature selection in terms of estimated model coefficients.

S4 Model Training

The multi-class logistic regression (see Section S1.4.3) classifiers described in the main article Sections Breast Cancer Subtyping and Colorectal Cancer Subtyping were trained according to the following procedure. After pre-processing (Sections S1.2 and S1.3), we used subtype-based stratified sampling to split each dataset, selecting 80% of the patient samples for training and 20% for testing. The training data was normalized using min-max normalization to prevent larger-scale features from dominating the feature selection process and the classification. Test samples were normalized using the training min-max statistics.

We first used 5-fold cross-validation (CV) to tune the parameter λ of a standard logistic regression

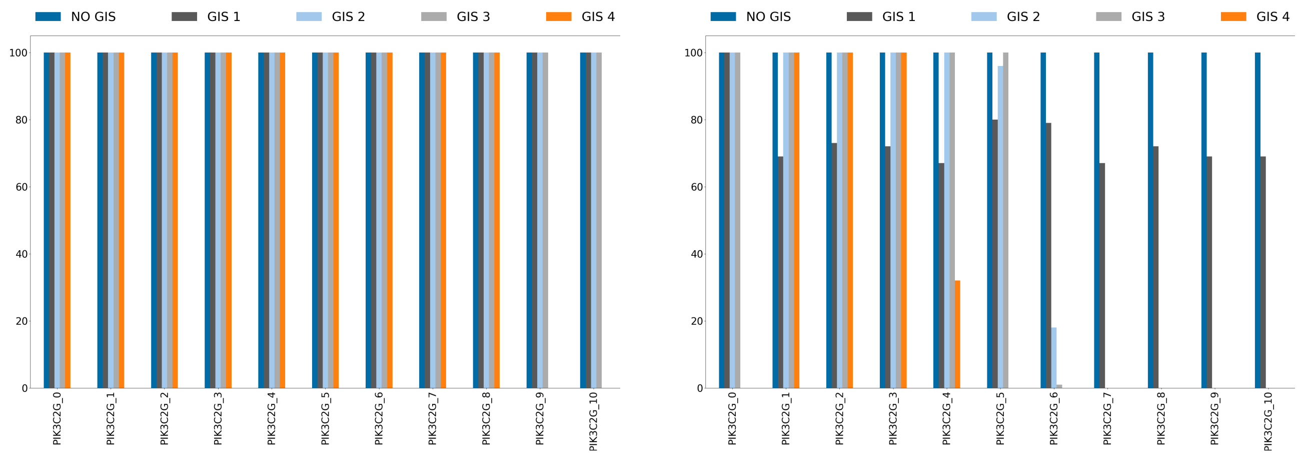

(a) *PIK3C2G* ($\lambda = 0.20$)   (b) *PIK3C2G* ($\lambda = 1.0$)

Figure S3: Multicollinearity analysis results for $\lambda = 0.20$ and $\lambda = 1.0$ for the *PIK3C2G* gene. Each bar indicates how many times each feature, the original one or a copy of it, is selected across 100 repetitions. Bars are grouped by feature: In each diagram, the first set of bars on the left refer to the original feature, and the remaining 10 sets refer to its copies.

Table S4: Cross-validation results.

Dataset	Breast Cancer					Colorectal Cancer				
λ	N	Accuracy	Recall	Precision	F1-score	N	Accuracy	Recall	Precision	F1-score
0.5	671	0.91669	0.91770	0.89761	0.90485	1,206	0.79030	0.77064	0.78813	0.77027
1.0	376	0.91420	0.91422	0.89536	0.90241	675	0.78626	0.76630	0.78184	0.76519
**2.0**	**194**	**0.90425**	**0.91802**	**0.88695**	**0.89857**	**370**	**0.76806**	**0.74785**	**0.75966**	**0.74311**
2.5	158	0.90230	0.91933	0.88798	0.89916	301	0.75794	0.73473	0.74559	0.72909
5.0	90	0.87813	0.89707	0.85742	0.87103	127	0.73170	0.71719	0.72007	0.71190

N is the number of features selected.
Selected λ value and corresponding performance are in bold.

with LASSO regularization over a range of values that can substantially reduce the huge dimensionality of the original feature space. We used accuracy as the main evaluation metric. However, due to the high-class imbalance present in both datasets, we also analyzed precision, recall, and F1-score values to ensure that the accuracy value would provide a true picture of the model performance across all the different classes. Cross-validation results on the Breast Cancer and Colorectal Cancer datasets are shown in Table S4. We selected a $\lambda = 2.0$ for both datasets as it provided good results in terms of classification performance, while simultaneously reducing the original dimensionality of the feature space.

Then, we trained both the standard and *GIS*-weighted LASSO regularized models on 10 different permutations of all training samples. We evaluated both models on 5-fold cross-validation and theNima Dehmamy same test set to assess the robustness of the results to the order of the training samples, in terms of both classification performance and selected feature subsets. We used the accuracy (mean and standard deviation across the 10 training set sample permutations) as the main performance evaluation metric to assess and compare the two classification models, along with the stability of the selected feature subsets (their union and intersection across the 10 permutations). Results are reported in Table 2 in the main article. We also computed the recall, precision, and F1-score to verify that the accuracy metric properly reflected the classifier's performance. Their values are reported in Table S5 and Table S6 for the BRCA and CRC test sets, respectively.

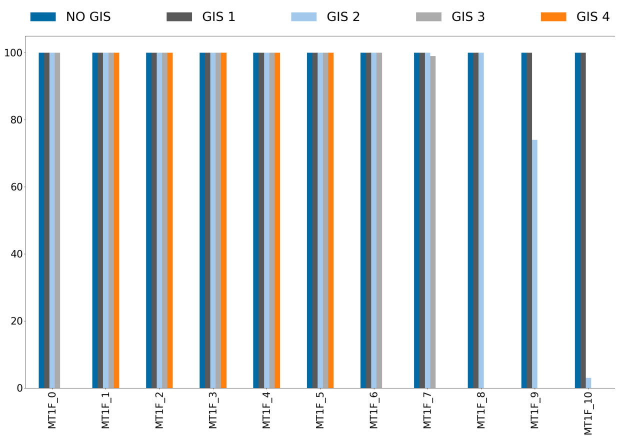

(a) *MT1F* ($\lambda = 0.25$)

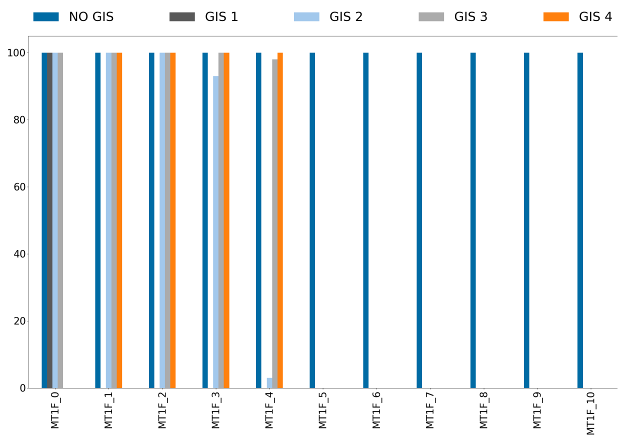

(b) *MT1F* ($\lambda = 0.50$)

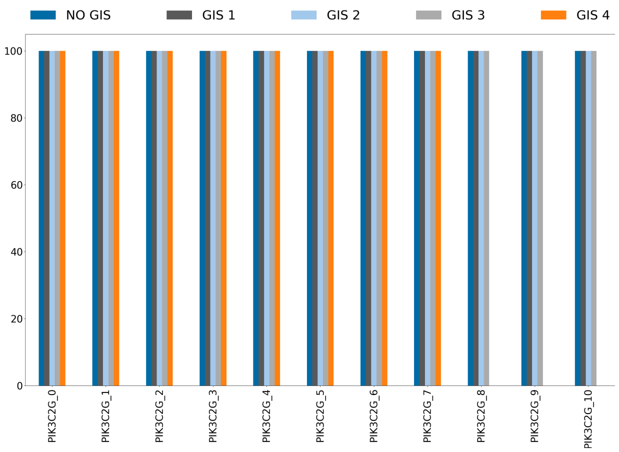

(c) *PIK3C2G* ($\lambda = 0.25$)

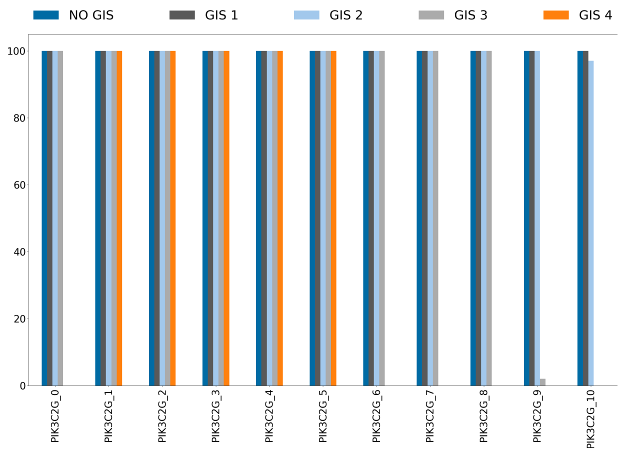

(d) *PIK3C2G* ($\lambda = 0.50$)

Figure S4: Multicollinearity analysis results for $\lambda = 0.25$ and $\lambda = 0.50$ for genes *MT1F* and *PIK3C2G*. Each bar indicates how many times each feature, the original one or a copy of it, is selected across 100 repetitions. Bars are grouped by feature: In each diagram, the first set of bars on the left refers to the original feature, and the remaining 10 sets refer to its copies.

S5 Biological Validation

We performed different types of evaluations to validate the effectiveness of our proposed approach in enhancing the biological interpretability of the results in the considered application use cases. We compared the feature/gene subsets selected by the standard LASSO and the *GIS*-weighted regularized models through the following analyses. The first analysis aimed at identifying the significantly enriched biological terms in the feature subsets selected by the two models, analyzing each subtype-specific subset separately. For any subtype-specific feature subset, we only considered the robust features, i.e., those from the intersection of the features obtained across the 10 training sample permutations evaluated. We used Fisher's Exact test to evaluate the statistically significant enrichment of GO terms, Reactome biological pathways, and HPO terms annotated to each selected feature subset with respect to the entire set of genes available to the model during training, i.e., the total number of genes in each dataset. The obtained p-values were adjusted for multiple testing using Benjamini-Hochberg false discovery rate (FDR) correction, and the significance level was set to 0.05.

To further characterize and assess the relevance of the features selected with our proposed approach, we extracted the subsets of genes selected only by the standard or only by the *GIS*-weighted LASSO regularization. These distinct subsets of genes were obtained by considering the union of the robust features (intersection across the 10 training sample permutations) selected across the differ-

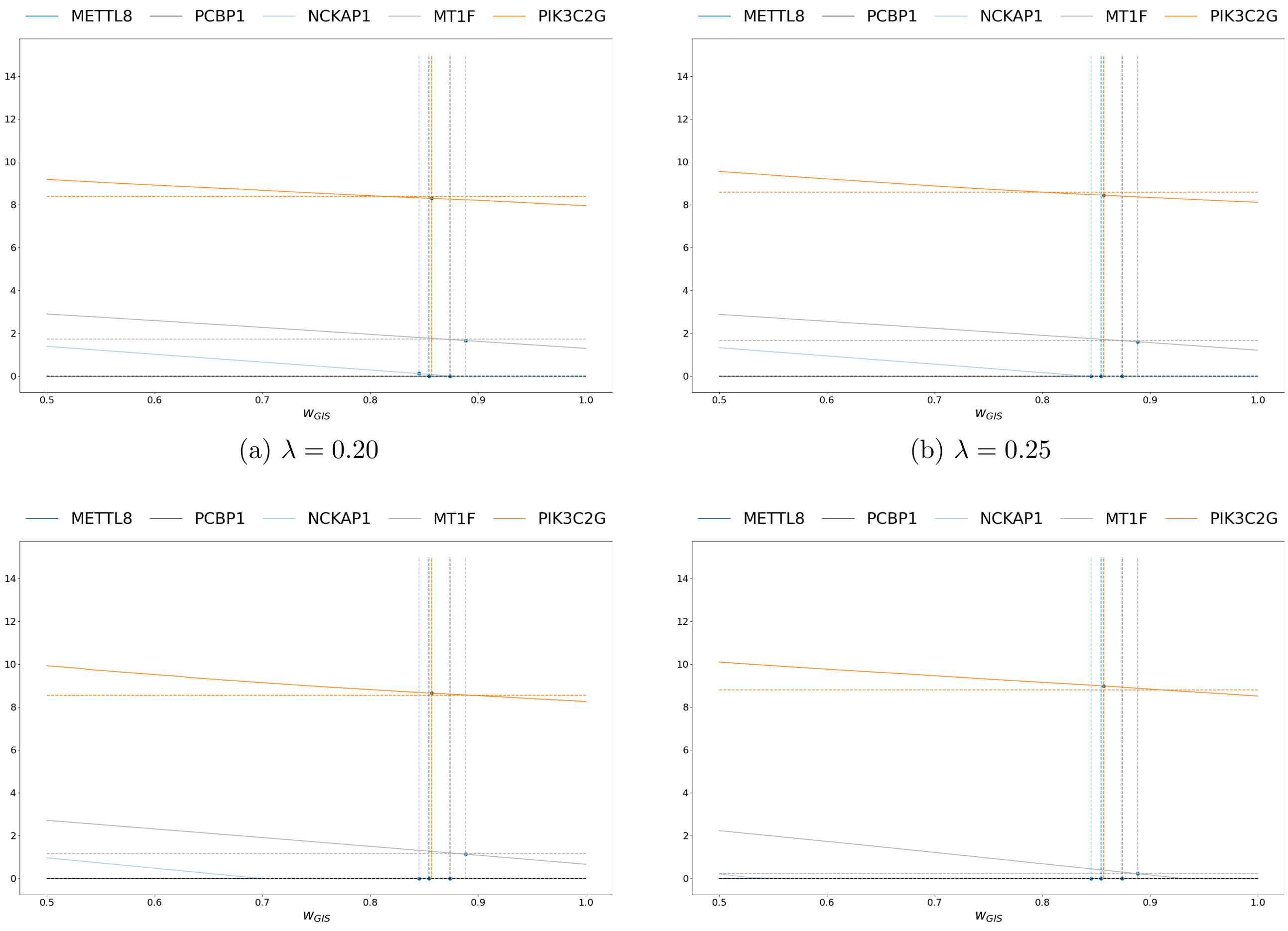

(a) $\lambda = 0.20$ (b) $\lambda = 0.25$

(c) $\lambda = 0.50$ (d) $\lambda = 1.0$

Figure S5: Predictive power analysis results. Parameter value estimates along the y-axis, w_{GIS} values in their entire range [0.5,1] along the x-axis. The horizontal dashed lines indicate the coefficient of the corresponding features for standard LASSO regularization. The vertical dashed lines indicate the original w_{GIS} values of the features. The intersection blue points indicate the corresponding feature coefficients for the original w_{GIS} values.

ent subtypes. We used DisGeNET [8], a knowledge database integrating information on gene-disease associations gathered from the scientific literature, curated databases, and Genome-Wide Association Studies (GWAS), to determine whether the only standard or only *GIS*-weighted LASSO selected genes belong to disease-related gene signatures. We also used MalaCards [9], a comprehensive and integrated database of human diseases and their annotations from multiple sources, including OMIM [10], DrugBank [11], Pubmed, and GeneCards [12], to extract Breast Cancer- and Colorectal Cancer-specific gene signatures, and evaluate their intersection with the subsets of only standard or only *GIS*-weighted LASSO selected genes. We additionally retrieved cancer-specific and disease-specific pathways from the KEGG database [13], an integrated resource providing information on the molecular functions of genes and their associated pathways, and computed the degree of overlap between the gene signatures associated with such pathways and the genes selected only by the standard or only by the *GIS*-weighted LASSO.

Table S5: Classification results (mean ± standard deviation) on the test set for BRCA subtyping.

Subtype	Recall_{LASSO}	Recall_{wGIS}	Precision_{LASSO}	Precision_{wGIS}	F1-score_{LASSO}	F1-score_{wGIS}
Basal	1.00000 (±0.0)	1.00000 (±0.0)	1.00000 (±0.0)	1.00000 (±0.0)	1.00000 (±0.0)	1.00000 (±0.0)
Her2	0.93750 (±0.0)	0.93750 (±0.0)	0.83333 (±0.0)	0.83333 (±0.0)	0.88235 (±0.0)	0.88235 (±0.0)
LumA	0.90826 (±0.0)	0.91743 (±0.0)	0.99000 (±0.0)	0.97087 (±0.0)	0.94737 (±0.0)	0.94340 (±0.0)
LumB	0.95238 (±0.0)	0.90476 (±0.0)	0.81633 (±0.0)	0.82609 (±0.0)	0.87912 (±0.0)	0.86364 (±0.0)
Overall	0.94953 (±0.0)	0.93992 (±0.0)	0.90991 (±0.0)	0.90757 (±0.0)	0.92721 (±0.0)	0.92235 (±0.0)

Table S6: Classification results (mean ± standard deviation) on the test set for CRC subtyping.

Subtype	Recall_{LASSO}	Recall_{wGIS}	Precision_{LASSO}	Precision_{wGIS}	F1-score_{LASSO}	F1-score_{wGIS}
CRIS A	0.84375 (±0.0)	0.84375 (±0.0)	0.90000 (±0.0)	0.87097 (±0.0)	0.87097 (±0.0)	0.85714 (±0.0)
CRIS B	0.81250 (±0.0)	0.75000 (±0.0)	0.86667 (±0.0)	0.85714 (±0.0)	0.83871 (±0.0)	0.80000 (±0.0)
CRIS C	0.87879 (±0.0)	0.87879 (±0.0)	0.93548 (±0.0)	0.96667 (±0.0)	0.90625 (±0.0)	0.92063 (±0.0)
CRIS D	0.90000 (±0.0)	0.90000 (±0.0)	0.72000 (±0.0)	0.72000 (±0.0)	0.80000 (±0.0)	0.80000 (±0.0)
CRIS E	0.73913 (±0.0)	0.78261 (±0.0)	0.73913 (±0.0)	0.75000 (±0.0)	0.73913 (±0.0)	0.76596 (±0.0)
Overall	0.83483 (±0.0)	0.83103 (±0.0)	0.83226 (±0.0)	0.83296 (±0.0)	0.83101 (±0.0)	0.82875 (±0.0)

S5.1 DisGeNET Findings

In this section, we present in Tables S7 and S8 the result from biological validation analyses performed to evaluate the degree of overlapping between the selected genes and disease-related gene signatures retrieved from DisGeNET.

S6 Additional Experiments

We performed additional experiments to compare and evaluate the performance of our proposed approach against several other feature selection methods. Since LASSO is an embedded feature selection method, we considered filter- and wrapper-based methods for these experiments. We also evaluated whether using an integrated approach to incorporate prior information would be better than using a filter-based approach. As prior knowledge, we used the proposed *Gene Information Score* to make approaches comparable in terms of prior information available.

S6.1 Feature Selection

We evaluated and compared the performance of various filter- and wrapper-based feature selection methods, both in terms of classification performance and biological interpretability. For filtering methods we considered Mutual Information (MI) [14, 15], Fisher's scores (FI) [16], minimum Redundancy - Maximum Relevance (mRMR) [17], Kolmogorov-Smirnov (KS) [18], and the Relief algorithm [19]. Instead, for the wrapped-based method we only considered forward feature selection (FS) as it provides more reliable estimates by adding features incrementally, allowing for complete model fitting at each step. Both Breast Cancer Subtyping and Colorectal Cancer Subtyping datasets described in Section S1 were processed as described in Section S4, and logistic regression was used as the classification model. We selected feature subsets with a comparable dimensionality k to the ones obtained with LASSO and *GIS*-weighted LASSO regularized models ($k = 200$ for BRCA, $k = 400$ for CRC). Because KS calculates per-class scores in a one-vs-rest scheme, we averaged the obtained scores across the different subtypes. We used accuracy as the performance evaluation metric but also computed the recall, precision, and F1-score to verify that the former metric properly reflected the classifier's

Table S7: Summary of the extracted DisGeNET signatures for BRCA subtyping.

DisGeNET Signature	Overlap_{LASSO}	Overlap_{wGIS}
Breast Carcinoma	5/4,963	18/4,963
Malignant Neoplasm of Breast	5/5,054	17/5,054
Triple Negative Breast Neoplasms	2/632	6/632
Carcinoma Breast stage IV	0/392	4/392
Invasive Carcinoma of Breast	1/385	3/385
Luminal A Breast Carcinoma	0/110	3/110
Luminal B Breast Carcinoma	1/80	2/80
HER2-positive Carcinoma of Breast	0/19	2/19
Advanced Breast Cancer	0/7	1/7
Secondary Malignant Neoplasm of female Breast	0/8	1/8
Breast Cancer (lobular)	0/22	1/22
Columnar Cell Hyperplasia of Breast	0/22	1/22
Columnar Cell Change of Breast	0/32	1/32
Neoplasm of uncertain or unknown behavior of Breast	0/44	1/44
Breast Diseases	0/51	1/51
Breast Fibrocystic Disease	0/75	1/75
Early-Stage Breast Carcinoma	0/76	1/76
Inflammatory Breast Carcinoma	0/102	1/102
Sporadic Breast Carcinoma	0/159	1/159
Breast Adenocarcinoma	0/168	1/168

Overlap_{LASSO}: number of the total 18 genes only selected by the standard LASSO regularization that are included in DisGeNET BRCA-specific gene signatures.

Overlap_{wGIS}: number of the total 39 genes only selected by the *GIS*-weighted LASSO regularization that are included in DisGeNET BRCA-specific gene signatures.

Table S8: Summary of the extracted DisGeNET signatures for CRC subtyping.

DisGeNET Signature	Overlap_{LASSO}	Overlap_{wGIS}
Colorectal Cancer	6/3,298	28*/3,298
Colorectal Carcinoma	6/2,931	27*/2,931
Colorectal Neoplasms	4/1,073	11/1,073
Colorectal Cancer metastatic	0/208	5/208
Colorectal Cancer recurrent	0/9	1/9
Hereditary Nonpolyposis Colorectal Carcinoma	0/26	1/26
Hereditary Nonpolyposis Colorectal Neoplasms	0/40	1/40

Overlap_{LASSO}: number of the total 38 genes only selected by the standard LASSO regularization that are included in DisGeNET CRC-specific gene signatures.

Overlap_{wGIS}: number of the total 86 genes only selected by the *GIS*-weighted LASSO regularization that are included in DisGeNET CRC-specific gene signatures.

*: Statistically significant percentage of selected genes included in DisGeNET CRC-specific gene signatures with respect to standard LASSO-selected ones. (Z-test for proportions, p-value < 0.05).

performance. To validate the biological interpretability of the selected feature subsets we followed the steps described in Section S5. Classification and biological validation results are reported in Tables S9 and S10 for BRCA and Tables S11 and S12 for CRC. In terms of classification performance, integrated approaches (LASSO, w_{GIS}) appeared to better classify BRCA patients into their corresponding subtypes, whereas mRMR appeared to work a bit better on the CRC dataset. Overall, the integrated approaches achieved higher classification performances, on average, compared to filter- and wrapper-based methods. Indeed, forward feature selection performed poorly, with a 6% and 7% decrease in accuracy on the test set. The performance of filter-based methods appeared to vary depending on the algorithm used. We computed the overlap between genes selected by filter-based methods and those chosen by the proposed *GIS*-weighted LASSO: it tends to be low (on average, 25.4% and 23.5% for the BCRA and CRC dataset, respectively). Indeed, there is a lack of consensus among the various feature selection algorithms concerning the selected features (N_{shared}), as each algorithm prioritizes different predictive characteristics. This difference is also evident in the results from the biological validation, which, however, are fairly similar on average. The *GIS*-weighted LASSO regularized model demonstrated to be quite robust across datasets, returning competitive performances in both scenarios. In contrast, other feature selection approaches, particularly the Relief algorithm, appeared to be more sensitive to dataset differences. Overall, the proposed *GIS*-weighted LASSO can consider feature interactions and dependencies in the feature selection process, achieving optimal classification performance while improving the biological interpretability of the results, particularly with respect to the standard LASSO algorithm.

S6.2 Feature Selection with Prior knowledge

We also performed experiments to test whether using an integrated approach to incorporate prior knowledge will be better than using a filter-based approach. We filtered genes based on their prior biological information, considering the proposed *GIS*. The BRCA and CRC datasets were processed as described in Section S4 and logistic regression was used as the classification model. As a baseline, we selected comparable feature subsets in terms of dimensionality ($k = 200$ for BRCA, $k = 400$ for CRC) only considering each gene's prior information, i.e., selecting the k genes with the highest *GIS* (prior_{all}). Additionally, we applied two filtering strategies that considered both prior biological knowledge and discriminative power. In the first approach, we initially selected k_{init} genes ($k_{init} = 500; 1,000$) based on their discriminative power (Fisher's scores) and then refined the selection by choosing k genes based on their biological relevance ($\text{prior}_{v1,k_{init}}$). Alternatively, we first filtered genes based on their biological relevance and then based on their discriminative power ($\text{prior}_{v2,k_{init}}$). Classification and biological validation results for these experiments are reported in Tables S9 and S10 for BRCA and Tables S11 and S12 for CRC. While the prior_{all} and prior_{v2} approaches demonstrated to be superior, as expected, in terms of the number of significant annotation terms retrieved and the overlap of selected genes with disease-specific or cancer-related gene signatures, both approaches significantly underperformed in classification accuracy on the test set compared to the proposed integrated approach. Additionally, although these methods selected more genes associated with relevant KEGG pathways, the overlap with MalaCards and DisGeNET for the BRCA dataset was much lower, suggesting that such filtering methods tend to retrieve more general knowledge. In contrast, the integrated approach appeared to focus gene selection more effectively on disease-specific genes, particularly on the BRCA dataset. On the other hand, prior_{v1} showed weaker classification performance while achieving comparable or lower biological validation results, particularly in the number of selected genes overlapping with known disease signatures. While the latter is somehow expected, as the discriminative power of genes mainly guides the selection, the lower prediction performance might indicate such filtering might be too restrictive. All this highlights that the proposed integrated approach not only excels in classification tasks but also in selecting features that are more biologically relevant to the study's objectives.

Table S9: Classification results on the test set for the additional experiments on the BRCA dataset.

Model	Accuracy	Recall	Precision	F1-score
LASSO	**0.93564**	**0.94953**	**0.90991**	**0.92721**
w_{GIS}	**0.93069**	**0.93992**	0.90757	**0.92235**
MI	0.91089	0.87011	0.90399	0.88248
FI	0.91584	0.92076	0.90571	0.91032
mRMR	0.92574	0.89311	0.89625	0.90311
KS	0.91089	0.89311	**0.91038**	0.90053
Relief	0.82673	0.80786	0.86273	0.83076
FS	0.87624	0.86608	0.87961	0.87159
prior_{all}	0.90099	0.88356	0.87513	0.87677
$\text{prior}_{v1,500}$	0.80198	0.76241	0.75813	0.75924
$\text{prior}_{v1,1000}$	0.82178	0.78361	0.80130	0.78832
$\text{prior}_{v2,500}$	0.88119	0.89739	0.85535	0.87208
$\text{prior}_{v2,1000}$	0.90594	0.91382	0.88255	0.89714

First and second best performance in bold.

Table S10: Biological validation results for the additional experiments on the BRCA dataset. Number of GO, KEGG or Reactome biological pathway, or HPO annotation terms retrieved for each selected feature subset of size N (and related number of significantly enriched terms, with FDR p-value < 0.05), and overlap degree between relevant gene signatures and each selected feature subset.

Model	N	N_{shared}	GO	KEGG	Reactome	HPO	KEGG BRCA	KEGG cancer	MalaCards	DisGeNET
LASSO	194	177	4,604 (195)	177 (0)	641 (25)	2,413 (2)	4	18	37	57
w_{GIS}	214	all	5,198 (354)	206 (0)	813 (32)	1,346 (3)	7	21	43	58
MI	200	51	4,305 (318)	139 (6)	532 (70)	2,886 (25)	9	22	39	49
FI	200	45	4,381 (178)	150 (5)	495 (47)	2,839 (1)	8	19	40	48
mRMR	200	53	4,476 (224)	150 (3)	535 (55)	3,008 (1)	9	20	46	55
KS	200	56	4,390 (412)	155 (7)	576 (119)	2,738 (49)	6	22	48	51
Relief	200	51	4,144 (221)	179 (7)	456 (28)	3,037 (117)	6	16	47	58
FS	200	8	4,036 (478)	162 (12)	548 (26)	2,597 (14)	3	11	17	46
prior_{all}	200	4	4,938 (710)	206 (30)	852 (78)	6,151 (4,833)	19	30	25	41
$\text{prior}_{v1,500}$	200	4	4,567 (599)	239 (69)	620 (122)	2,627 (0)	4	18	17	25
$\text{prior}_{v1,1000}$	200	4	4,355 (231)	186 (22)	568 (34)	2,339 (0)	2	10	11	15
$\text{prior}_{v2,500}$	200	9	5,958 (1,089)	243 (69)	850 (82)	6,050 (4,646)	15	40	37	48
$\text{prior}_{v2,1000}$	200	18	5,471 (709)	197 (12)	759 (48)	5,407 (3,799)	9	24	34	43

N_{shared}: number of selected genes shared with the proposed *GIS*-weighted LASSO (w_{GIS}).
KEGG BRCA: genes belonging to KEGG pathways related to BRCA.
KEGG cancer: genes belonging to KEGG pathways related to cancer.
MalaCards: genes belonging to BRCA MalaCards gene signatures.
DisGeNET: number of BRCA-related DisGeNET gene signatures retrieved.
w_{GIS} refers to *GIS*-weighted LASSO.

Table S11: Classification results on the test set for the additional experiments on the CRC dataset.

Model	Accuracy	Recall	Precision	F1-score
LASSO	**0.83871**	**0.83483**	0.83226	**0.83101**
w_{GIS}	**0.83871**	0.83103	0.83296	0.82875
MI	0.80645	0.78456	0.79846	0.78782
FI	**0.83871**	0.81951	0.83313	0.82365
mRMR	**0.85484**	**0.84584**	**0.85209**	**0.84716**
KS	0.79839	0.78228	0.78792	0.78388
Relief	0.82258	0.80500	**0.83613**	0.81320
FS	0.76613	0.74225	0.76109	0.74178
prior$_{all}$	0.75806	0.72875	0.73632	0.73073
prior$_{v1,500}$	0.78226	0.76861	0.77345	0.76823
prior$_{v1,1000}$	0.72581	0.69508	0.70884	0.69212
prior$_{v2,500}$	0.75806	0.72883	0.75366	0.73042
prior$_{v2,1000}$	0.79839	0.77315	0.81799	0.77364

First and second best performance in bold.

Table S12: Biological validation results for the additional experiments on the CRC dataset. Number of GO, KEGG or Reactome biological pathway, or HPO annotation terms retrieved for each selected feature subset of size N (and related number of significantly enriched terms, with FDR p-value $<$ 0.05), and overlap degree between relevant gene signatures and each selected feature subset.

Model	N	N$_{shared}$	GO	KEGG	Reactome	HPO	KEGG CRC	KEGG cancer	MalaCards	DisGeNET
LASSO	369	339	5,815 (3)	248 (1)	834 (0)	3,415 (0)	1	28	28	7
w_{GIS}	420	all	6,689 (21)	281 (1)	987 (0)	4,226 (0)	2	36	40	10
MI	400	76	6,271 (0)	274 (0)	1,000 (2)	3,809 (0)	1	30	32	7
FI	400	94	6,153 (0)	263 (0)	1,033 (7)	3,619 (0)	2	33	34	5
mRMR	400	126	6,081 (19)	250 (0)	928 (0)	3,824 (19)	2	32	35	7
KS	400	72	6,253 (0)	278 (1)	1,048 (1)	3,712 (0)	2	36	39	5
Relief	400	101	6,106 (466)	267 (20)	740 (55)	3,640 (39)	2	29	65	11
FS	400	13	5,878 (742)	238 (829)	735 (127)	3,611 (1)	1	24	21	9
prior$_{all}$	400	21	7,806 (1,613)	266 (112)	1,250 (237)	7,435 (5,695)	11	66	48	14
prior$_{v1,500}$	400	12	6,052 (949)	261 (92)	777 (178)	3,667 (0)	3	30	21	8
prior$_{v1,1000}$	400	7	5,684 (586)	258 (71)	814 (131)	3,417 (0)	3	21	16	9
prior$_{v2,500}$	400	27	8,049 (1,736)	272 (135)	1,307 (292)	7,404 (5,644)	13	76	50	14
prior$_{v2,1000}$	400	33	8,672 (2,107)	299 (153)	1,287 (225)	7,298 (5,338)	12	85	49	14

N$_{shared}$: number of selected genes shared with the proposed *GIS*-weighted LASSO (w_{GIS}).
KEGG CRC: genes belonging to KEGG pathways related to CRC.
KEGG cancer: genes belonging to KEGG pathways related to cancer.
MalaCards: genes belonging to CRC MalaCards gene signatures.
DisGeNET: number of CRC-related DisGeNET gene signatures retrieved.
w_{GIS} refers to *GIS*-weighted LASSO.

References

[1] J N Weinstein, E A Collisson, G B Mills, et al. The cancer genome atlas pan-cancer analysis project. *Nature Genetics*, 45(10):1113–1120, 2013.

[2] N A O'Leary, M W Wright, J R Brister, et al. Reference sequence (RefSeq) database at NCBI: current status, taxonomic expansion, and functional annotation. *Nucleic Acids Research*, 44(D1):D733–D745, 2016.

[3] J S Parker, M Mullins, M C U Cheang, et al. Supervised risk predictor of breast cancer based on intrinsic subtypes. *Journal of Clinical Oncology*, 27(8):1160–1167, 2009.

[4] Y Hoshida. Nearest template prediction: a single-sample-based flexible class prediction with confidence assessment. *PloS One*, 5(11):e15543, 2010.

[5] M Ashburner, C A Ball, J A Blake, et al. Gene ontology: tool for the unification of biology. The Gene Ontology Consortium. *Nature Genetics*, 25(1):25–29, 2000.

[6] B Jassal, L Matthews, G Viteri, et al. The reactome pathway knowledgebase. *Nucleic Acids Research*, 48(D1):D498–D503, 2019.

[7] P N Robinson, S Köhler, S Bauer, et al. The Human Phenotype Ontology: a tool for annotating and analyzing human hereditary disease. *The American Journal of Human Genetics*, 83(5):610–615, 2008.

[8] J Piñero, À Bravo, N Queralt-Rosinach, et al. DisGeNET: a comprehensive platform integrating information on human disease-associated genes and variants. *Nucleic Acids Research*, 45(D1):D833–D839, 2016.

[9] N Rappaport, M Twik, I Plaschkes, et al. MalaCards: an amalgamated human disease compendium with diverse clinical and genetic annotation and structured search. *Nucleic Acids Research*, 45(D1):D877–D887, 2016.

[10] A Hamosh, A F Scott, J Amberger, et al. Online Mendelian Inheritance in Man (OMIM), a knowledgebase of human genes and genetic disorders. *Nucleic Acids Research*, 30(1):52–55, 2002.

[11] D S Wishart, C Knox, A C Guo, et al. DrugBank: a comprehensive resource for in silico drug discovery and exploration. *Nucleic Acids Research*, 34:D668–D672, 2006.

[12] G Stelzer, N Rosen, I Plaschkes, et al. The GeneCards Suite: From gene data mining to disease genome sequence analyses. *Current Protocols in Bioinformatics*, 54(1):1.30.1–1.30.33, 2016.

[13] M Kanehisa and S Goto. KEGG: kyoto encyclopedia of genes and genomes. *Nucleic Acids Research*, 28(1):27–30, 2000.

[14] C E Shannon. A Mathematical Theory of Communication. *The Bell System Technical Journal*, 27:379–423, 1948.

[15] R Battiti. Using Mutual Information for Selecting Features in Supervised Neural Net Learning. *IEEE Transactions on Neural Networks*, 5:537 – 550, 08 1994.

[16] R O Duda and P E Hart and D G Stork. *Pattern Classification.* Wiley, New York, 2 edition, 2001.

[17] Ding, C and Peng, H. Minimum Redundancy Feature Selection From Microarray Gene Expression Data. *Journal of Bioinformatics and Computational Biology*, 3:523– 528, 09 2003.

[18] J Biesiada and W Duch. Feature Selection for High-Dimensional Data - A Pearson Redundancy Based Filter. *Computer Recognition Systems 2*, pages 242–249, 2007.

[19] K Kira and L A Rendell. The feature selection problem: traditional methods and a new algorithm. In *Proceedings of the Tenth National Conference on Artificial Intelligence*, page 129–134. AAAI Press, 1992.
